# Nonagenarians admission and prognosis in a tertiary center intensive coronary care unit – a prospective study

**DOI:** 10.1186/s12877-023-03851-z

**Published:** 2023-03-20

**Authors:** Sharon Bruoha, Tomer Maller, Ranel Loutati, Nimrod Perel, Meir Tabi, Louay Taha, Chaim Yosefy, Jamal Jafari, Omri Braver, Itshak Amsalem, Rafael Hitter, Mohamed Manassra, Nir Levy, Ismael Abu-Alkean, Kamal Hamyil, Yoed Steinmetz, Hani Karameh, Mohamed Karmi, David Marmor, Arik Wolak, Michael Glikson, Elad Asher

**Affiliations:** 1 Department of Cardiology, Barzilai Medical Center, The Ben-Gurion University of the Negev, Hahistadrout 2, Ashkelon, 7830604 Israel; 2 Jesselson Integrated Heart Center, Faculty of Medicine, Shaare Zedek Medical Center, Hebrew University of Jerusalem, Jerusalem, Israel

**Keywords:** Nonagenarians, Cardiac intensive care, Acute coronary syndrome

## Abstract

**Background:**

With increasing life expectancy, the prevalence of nonagenarians with cardiovascular disease is steadily growing. However, this population is underrepresented in randomized trials and thus poorly defined, with little quality evidence to support and guide optimal management. The aim of the present study was to evaluate the clinical management, therapeutic approach, and outcomes of nonagenarians admitted to a tertiary care center intensive coronary care unit (ICCU).

**Methods:**

We prospectively collected all patients admitted to a tertiary care center ICCU between July 2019 – July 2022 and compared nonagenarians to all other patients. The primary outcome was in-hospital mortality.

**Results:**

A total of 3807 patients were included in the study. Of them 178 (4.7%) were nonagenarians and 93 (52%) females. Each year the prevalence of nonagenarians has increased from 4.0% to 2019, to 4.2% in 2020, 4.6% in 2021 and 5.3% in 2022. Admission causes differed between groups, including a lower rate of acute coronary syndromes (27% vs. 48.6%, p < 0.001) and a higher rate of septic shock (4.5% vs. 1.2%, p < 0.001) in nonagenarians. Nonagenarians had more comorbidities, such as hypertension, renal failure, and atrial fibrillation (82% vs. 59.6%, 23% vs. 12.9%, 30.3% vs. 14.4% p < 0.001, respectively). Coronary intervention was the main treatment approach, although an invasive strategy was less frequent in nonagenarians in comparison to younger subjects. In-hospital mortality rate was 2-fold higher in the nonagenarians (5.6% vs. 2.5%, p = 0.025).

**Conclusion:**

With increasing life expectancy, the prevalence of nonagenarians in ICCU’s is expected to increase. Although nonagenarian patients had more comorbidities and higher in-hospital mortality, they generally have good outcomes after admission to the ICCU. Hence, further studies to create evidence-based practices and to support and guide optimal management in these patients are warranted.

## Background

Over the last several decades, life expectancy in western countries has improved substantially [1]. Longer lifespan has been associated with increased risk of age-associated diseases, including cardiovascular disease (CVD) which remains the leading cause of death in higher age groups. Therefore, we expect to see more nonagenarians with CVD including acute coronary syndromes (ACS), valvular disease, heart failure (HF), cardiac arrhythmias, and venous thromboembolism (VTE) in the near future [2]. However, the unique characteristics of the very elderly render their management particularly challenging. First, elderly patients may present with atypical symptoms during ACS leading to delayed diagnosis and administration of therapy [3]. Second, nonagenarians often have multiple comorbidities [4], significant frailty [5], and varying degrees of cognitive impairment [6]. Consequently, doubts regarding futility of care often arise that can lead to under-treatment of this patient population [7]. Moreover, age-related vascular degeneration such as atherosclerosis, arterial calcification and endothelial dysfunction [2, 8], responsible for the high incidence of ischemic heart disease, are also closely related to a more complex coronary interventions, concomitant presence of peripheral vascular disease, and increased susceptibility to procedure related complications [9]. This, together with the vulnerability of the elderly to medication toxicity, drug-drug interactions, and side-effects (e.g., bleeding, low blood pressure, renal failure) may limit the use of effective therapies [10, 11] including revascularization [12, 13]. Hence, nonagenarians have been poorly represented in randomized trials, which has resulted in limited evidence-based data and long-term follow-up to guide tailored management.

The aim of the current study was to evaluate the clinical management, therapeutic approach, and outcomes of nonagenarians admitted to a tertiary care center intensive coronary care unit (ICCU).

## Methods

All patients aged ≥ 90 years admitted to a tertiary care ICCU at Shaare Zedek Medical Center, Israel, between July 2019 – October 2022 were prospectively included.

Data were prospectively and anonymously documented in an electronic case report form (eCRF). Data were checked for accuracy and out-of-range values by the study coordinator.

Demographic data, presenting symptoms, ECG, comorbid conditions and physical examination were systematically recorded. Laboratory, imaging, angiographic results, and clinical course data were collected as well.

Patients were followed up to one year after discharge. Patients were stratified according to the initial diagnosis (ACS, Valvular disease, Arrhythmia, HF, and VTE). Patients with ACS and acute valvular disease were further stratified according to the treatment strategy (invasive vs. conservative).

In patients with ACS, invasive or conservative strategy was chosen according to the discretion of the treating senior cardiologist and were in accordance with the European Society of Cardiology (ESC) guidelines for ACS. The diagnosis of myocardial infraction was based on symptoms of myocardial ischemia, new ECG ischemic changes, and a rising and/or falling pattern of high-sensitivity troponin with at least one value above the 99th percentile URL. ST-segment elevations myocardial infarction (STEMI) and non-ST-segment elevations myocardial infarction (NSTEMI) were defined according to the ESC guidelines for ACS [14, 15]. All patients underwent at least one transthoracic echocardiography (TTE) exam to evaluate the left ventricular ejection fraction (LVEF) and valvular function. The diagnosis of HF with preserved EF vs. mildly reduced EF vs. reduced EF was based on the ESC guidelines HF diagnostic criteria [16]. Transesophageal echocardiography (TEE) or invasive hemodynamic studies were performed in selected cases with diagnostic uncertainty. Computed tomography with contrast was utilized to evaluate for presence of pulmonary embolism (PE).

The Institutional review board approved the study on the basis of strict maintenance of participant anonymity with de-identified data during database analysis. No individual consent was obtained. The authors have no conflicts of interest to declare. No funding was used for the study. All methods were performed in accordance with the relevant guidelines and regulations.

We compared demographic and clinical characteristics, hospital admission causes, complications, and mortality between Nonagenarians and the younger patients. Mortality rates were derived from The Israeli Ministry of Internal Affairs medical record database, which is continuously updated by every medical center in Israel.

### Statistical analysis

Patients’ characteristics were presented as numbers (%) for categorical variables, and as means (SD) or medians (IQR) for normal and non-normal distributed continuous variables respectively. Comparison of categorical variables was done by Chi-squared test and Fisher’s exact tests. Student T-Test and Mann-Whitney tests were performed for comparison of normally and non-normally distributed continuous variables, respectively.

Risk factors for mortality were found by applying univariate logistic model between categorical variables and the in-hospital mortality outcome. All tests were two-sided. P < 0.05 was considered statistically significant. Analyses were carried out using R software, Version 4.2.0 (R Foundation for Statistical Computing).

## Results

### Patients’ characteristics

A total of 3807 patients were included in the study. Of them 178 (4.7%) were nonagenarians and 93 (52%) were female. Patient characteristics are presented in Table 1.

**Table 1:** Patients’ characteristics

Clinical Variables	All patients (n = 3807)	Nonagenarians (n = 178) (4.7%)	Younger patients (n = 3629) (95.3%)	P-Value
Age in years (mean ± SD)	66.9 ± 16	92.5 ± 2.5	66 ± 15	< 0.001
Female sex – no. (%)	1171 (30.7%)	93 (52.2%)	1078 (29.7%)	< 0.001
BMI mean (SD)	-	25.6 (5.77)	28 (5.35)	< 0.001
Cr mean (SD)	-	1.30 (0.753)	1.31 (3.89)	1
Hypertension	2310 (60.6%)	146 (82%)	2164 (59.6%)	< 0.001
DM	1399 (36.7%)	37 (20.8%)	1362 (37.5%)	< 0.001
Hyperlipidemia	1980 (52%)	88 (49.4%)	1892 (52.1%)	0.531
Smoking	1018 (27%)	2 (1%)	1016 (28%)	< 0.001
Prior CAD	1162 (30.5%)	60 (33.7%)	1102 (30.4%)	0.389
Prior CABG	240 (6.3%)	7 (3.9%)	233 (6.4%)	0.238
CVA	279 (7.3%)	21 (11.8%)	258 (7.1%)	0.028
PAD	166 (4.3%)	7 (3.9%)	159 (4.4%)	0.922
CHF or CMP	625 (16.4%)	52 (29.2%)	573 (15.8%)	< 0.001
EF % mean (SD)	-	53.5 (12.7)	50.9 (14.1)	0.01
COPD	309 (7.8%)	7 (3.9%)	302 (8.3%)	0.05
Atrial fibrillation	575 (15%)	54 (30.3%)	521 (14.4%)	< 0.001
Anemia	200 (5%)	16 (9%)	184 (5.1%)	0.0344
CKD	509 (13%)	41 (23%)	468 (12.9%)	< 0.001
Cognitive Decline	129 (3.3%)	20 (11.2%)	109 (3%)	< 0.001
Debilitated*	136 (3.5%)	28 (15.7%)	108 (3%)	< 0.001
Albumin g\dL (SD)	-	3.45 (0.536)	3.72 (1.217)	**< 0.001**

BMI = Body mass index; Cr = Creatinine; GFR = Glomerular filtration rate; DM = Diabetes mellitus; CAD = coronary artery disease; CABG = Coronary artery bypass graft surgery; CVA = Cerebrovascular accident; PAD = Peripheral artery disease; CHF = Congestive heart failure; CMP = Cardiomyopathy; EF = Ejection fraction; COPD = Chronic obstructive pulmonary disease; HTN = Hypertension; CKD = chronic kidney disease. * Patients were defined as debilitated if they had at least one impairment in physical activities of daily living such as walking or eating

**Fig. 1:**
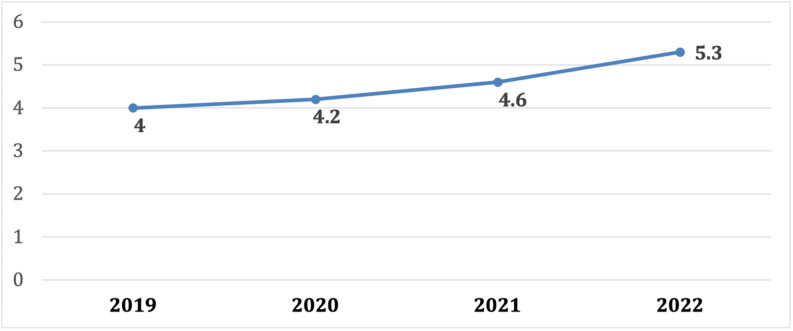
ICCU admission rate (%) in Nonagenarians between 2019 and 2022 ICCU – Intensive cardiac care unit

The prevalence of nonagenarians has increased from 4.0% to 2019, to 4.2% in 2020, 4.6% in 2021, and 5.3% in 2022 (Fig. 1). Mean age in the Nonagenarians was 92.5 (± 2.5) years old vs. 66 (± 15) years old in the younger patients (p < 0.001). Not surprisingly, nonagenarians had more comorbidities, such as hypertension, chronic kidney disease, and atrial fibrillation (82% vs. 59.6%, 23% vs. 12.9%, 30.3% vs. 14.4% p < 0.001, respectively), whereas diabetes and smoking were more common in the younger patients (37.5% vs. 20.8% and 28% vs. 1% p < 0.001, respectively). Importantly, cognitive decline affected 20 (11%) of the nonagenarians as compared with 109 (3%) of the younger patients (p < 0.05). Overall, 48 nonagenarians presented with ACS (including STEMI and NSTEMI) with PCI (either primary or non-primary) performed in 38 patients (80%). In contrast, 1766 cases of ACS were reported in the younger subjects with PCI performed in 1606 patients (90%). The rate of invasive treatment was significantly different between groups (p = 0.005).

### Admission diagnoses

Diagnoses on admission differed between groups, with a lower rate of ACS (27% vs. 48.6%, p < 0.001) and arrhythmias (4.5% vs. 10.3%, p = 0.017) and a higher rate of septic shock (4.5% vs. 1.2%, p < 0.001) and post-procedure status (30% vs. 18%, p < 0.001) in the nonagenarians.

Interestingly, one of the most common indications for ICCU admission was post-intervention management. Transcatheter aortic valve implantation (TAVI), permanent pacemaker implantation, and mitral transcatheter edge-to-edge repair (TEER) were the most frequently performed procedures as shown in Table 2.

**Table 2:** Indications for ICCU admissions

Indication for admission	All patients (n = 3807)	Nonagenarians (n = 178) (4.7%)	Younger patients (n = 3629) (95.3%)	P-Value
ACS	1814 (47.5%)	48 (27%)	1766 (48.6%)	< 0.001
STEMI	1014 (26.6%)	23 (12.9%)	991 (27.3%)	< 0.001
NSTEMI	800 (21%)	25 (14%)	775 (21.4%)	0.025
CHF	201 (5.3%)	7 (4%)	194 (5.3%)	0.515
Cardiogenic Shock	256 (6.7%)	13 (7.3%)	243 (6.7%)	0.871
Septic Shock	52 (1.3%)	8 (4.5%)	44 (1.2%)	< 0.001
Arrhythmia	380 (10%)	8 (4.5%)	372 (10.3%)	0.017
PE	152 (4%)	7 (4%)	145 (4%)	0.9
Post procedure	711 (18.5%)	52 (30%)	659 (18%)	< 0.001
TAVI	297 (7.8%)	31 (18%)	266 (7.3%)	< 0.001
PPM	69 (1.8%)	7 (4%)	63 (1.7%)	0.33
LAO	19 (0.5%)	1 (0.5%)	18 (2.2%)	0.9
TEER	51 (1.2%)	5 (3%)	46 (1.3%)	0.08
EP	135 (3.5%)	6 (3.3%)	129 (3.5%)	0.89
Other	72 (10%)	2 (4%)	70 (10.5%)	0.62
CPR	146 (3.8%)	9 (5.1%)	137 (3.8%)	0.503

ACS = Acute coronary syndrome; STEMI = ST elevation myocardial infarction; NSTEMI = non-ST-elevation myocardial infarction; CHF = congestive heart failure; TAVI = Transcatheter aortic valve implantation; PPM = Permanent pacemaker; LAO = Left atrial appendage occlusion; TEER = Transcatheter edge to edge repair; EP = Electrophysiology; CPR = Cardiopulmonary resuscitation

### Procedures and complications during admission

The type of procedures performed during the ICCU admission course are reported in Table 3. Invasive coronary intervention rates for ACS were lower among nonagenarians as compared with younger patients [38/48 (79%) vs. 1606/1765 (91%), respectively, p < 0.05]. Complication rate in the nonagenarians’ patients was significantly higher when compared with the younger patients [60 (33.5%) vs. 842 (23%), p < 0.01], as shown in Table 4. Acute renal failure (ARF) was the most frequent complication involving 10% (17) of the nonagenarian’s patients compared with only 4% (150) of the younger patients (p = 0.001).

**Table 3:** In-hospital interventions

Intervention	All patients (n = 3807)	Nonagenarians (n = 178) (4.7%)	Younger patients (n = 3629) (95.3%)	P-Value
Arterial line	704 (18.5%)	43 (24.2%)	661 (18.2%)	0.058
Pacemaker	119 (3%)	27 (15.1%)	92 (2.5%)	0.349
Non-primary PCI	683 (18%)	19 (10.7%)	664 (18.3%)	0.013
Primary PCI	961 (25%)	19 (10.7%)	942 (25.6%)	< 0.001
Total PCI	1644 (43%)	38 (21%)	1606 (44%)	< 0.001
Mechanical ventilation	313 (8%)	5 (3%)	308 (8.5%)	0.677
Vasopressors	494 (13%)	23 (13%)	471 (13%)	1
Hemodialysis	99 (2.5%)	18 (10%)	81 (2.2%)	< 0.001
Blood transfusion	105 (2.7%)	10 (5.6%)	95 (2.6%)	0.845

PCI = Percutaneous coronary intervention.

**Table 4:** In-hospital course and complications

Complication	All patients (n = 3807)	Nonagenarians (n = 178) (4.7%)	Younger patients (n = 3629) (95.3%)	P-Value
ARF	167 (4.3%)	17 (9.6%)	150 (4.1%)	0.001
Malignant arrhythmia	94 (2.5%)	2 (1.1%)	92 (2.5%)	0.35
CHF	144 (4%)	9 (5.1%)	135 (3.7%)	0.48
Mechanical complication	20 (0.5%)	0 (0%)	20 (0.6%)	0.64
Shock	194 (5%)	9 (5.1%)	185 (5.1%)	1
Sepsis	77 (2%)	7 (3.9%)	70 (1.9%)	0.11
Anoxic brain damage	14 (0.3%)	0 (0%)	14 (0.4%)	0.85
Significant bleeding	150 (4%)	11 (6%)	139 (4%)	0.171
Stroke or TIA	42 (1%)	5 (3%)	37 (1%)	0.062
Total Complications	902 (23.5%)	60 (33.5%)	842 (23%)	0.06

ARF = Acute renal failure; CHF = congestive heart failure; TIA = Transient ischemic attack.

### Length of admission

Interestingly, the mean length of admission was similar in both groups: 2.46 days for nonagenarians vs. 2.41 days for younger patients (p = 0.37).

### Follow up

Median follow up time was 310 days. 483 patients were lost during follow-up. 24 of them nonagenarians.

### Mortality rate

**Fig. 2:**
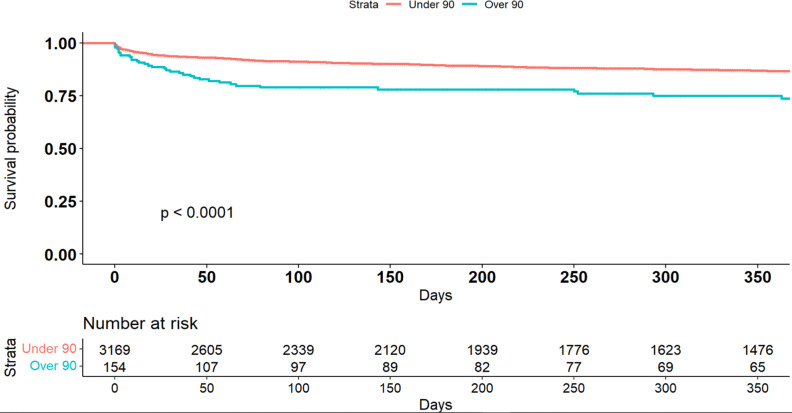
Kaplan–Meier survival curve for patients over 90 years old and patients under 90 years old Survival from Intensive coronary care unit (ICCU) admissions. Kaplan–Meier survival curve: patients over 90 years old (*blue*) and patients under 90 years old (*orange*). Groups were compared via Log-rank test (p < 0.0001)

In-hospital mortality rate was 2-fold higher in the nonagenarians as compared with the younger patients (5.6% vs. 2.5%, p = 0.025). Factors associated with in-hospital mortality were diagnosis of cardiogenic shock [HR = 6.76, 95% CI (1.23–29.71), p = 0.004] and prior TAVI [HR = 6.76, 95% CI (1.23–29.71), p = 0.004]. Long term survival (Fig. 2) was available for 3324 patients, including 154 nonagenarians. At follow-up, 310 days after discharge, 46 nonagenarians (30%) and 448 patients under 90 (14%) died (p < 0.0001). Factors associated with long term mortality were background of pulmonary hypertension [HR = 3.46, 95% CI (1.18–10.5), p = 0.014] and diabetes mellitus [HR = 2.76, 95% CI (1.21–6.31), p = 0.011]. However, when adjusted for age, mortality rate among nonagenarians and patients under 90 did not differ significantly (p = 0.2).

## Discussion

The present study evaluated characteristics and clinical outcomes among nonagenarians admitted to a tertiary care center ICCU over a 4-year time period. Our main findings were: (1) the prevalence of nonagenarians has increased over recent years; (2) nonagenarian patients had a different pattern of admission causes and and higher in-hospital mortality than younger subjects; (3) most nonagenarians presenting with ACS underwent coronary interventions although less than younger subjects; and (4) with high treatment intensity, the outcome in nonagenarians admitted to ICCU was better than expected in comparison with previously published clinical series.

The increase in longevity and the prevalence of elderly patients with cardiovascular diseases has translated into higher hospitalization rates for advanced age groups [17], with more nonagenarians occupying intensive care beds [18]. In the current study, nonagenarians accounted for ~ 5% of total admissions to our ICCU. Furthermore, we noticed a small but persistent annual growth of 0.2–0.7% of nonagenarian admissions during a 4-year period (Fig. 1).

Multimorbidity (≥ 2 simultaneously active chronic conditions) is almost universal among the elderly accounting for approximately 80% of patients over 75 years old [19]. The high comorbidity burden not only contribute to poor outcomes [20], but also limits the use of more aggressive therapies with higher risk for complications. Nevertheless, contemporary evidence that specifically included nonagenarians with ACS has shown benefits of an early invasive approach [21, 22].

Hypertension, chronic kidney disease, and atrial fibrillation, alone or in combination, were the most frequent chronic medical conditions seen in the older cohort. However, despite multiple comorbidities and potentially higher risk for adverse events in the nonagenarians, invasive coronary interventions were performed in the majority of the nonagenarian patients. Although less PCIs were performed in nonagenarians compared to younger patients, most nonagenarians underwent coronary interventions. However, is spite of presumably being a selected sample, nonagenarians admitted to ICCU for ACS still have a lower proportion of invasive interventions, presumably due to comorbidity and greater cognitive and functional decline. Radial approach PCIs [23], low contrast procedures [24] and less aggressive [25] antithrombotic regimens lead to safer coronary interventions and may favor an invasive strategy in high risk patients. With increasing life expectancy and the number of nonagenarians in a relatively good physical and mental health, the invasive approach might become more common.

We feel that treatment intensity was probably not associated with higher incidence of acute renal failure (9.6%) or major bleeding (6%), as similar incidence of these complications has been seen in other studies. For instance, a study evaluating 145 nonagenarians presenting with STEMI and managed invasively reported ARF and significant bleeding in 10% and 4% of patients, respectively [26].

The emergence of low-risk percutaneous valvular interventions has made this therapeutic option available for patients at high surgical risk with more nonagenarians undergo minimally invasive interventions. Importantly, in a substantial proportion (30%) of nonagenarians the indication for ICCU admission was post-procedure care after transcatheter valve interventions and implantation of pacemakers whereas the same indication for admission was noted in only 18% of the younger cohort, reflecting a relatively high complications rate with advanced age (p < 0.001). With the progressive gain of experience and availability of transcatheter solutions, the number of treated patients at advanced age will most likely continue to increase.

We report a in-hospital mortality rate of 5% in the older cohort compared to 2.5% in the younger patients. Previous studies have shown that nonagenarians with ACS have unfavorable outcomes with reported short-term mortality rates between 15%-20% [27–30]. However, in those studies primary PCI was less utilized, and a high proportion of patients received conservative management [31]. Due to the limited size of the samples it is impossible to conclude whether our aggressive approach to achieve prompt reperfusion with invasive therapies in most of our patients was beneficial and responsible for the relatively favorable short-term outcomes. Of note, in our study, coronary revascularization was achieved within 90 min in over 90% of patients presenting with STEMI; of those, approximately 80% were performed via radial access. In the present study, long term (310 days) survival was significantly higher in patients under 90 years.

The main limitation to our study is probably selection bias. In most cases, the nonagenarian patients occupying our ICCU beds were likely less disabled and had less cognitive impairment than other nonagenarians. Nevertheless, due to the small number of internal medicine wards in our medical center (4 wards), it is common practice to admit nonagenarians with cardiac problems in ICCU. Very elderly patients with poor cognitive and/or physical status are generally treated conservatively in other settings. Thus, our results may not apply to the general population of very elderly presenting with acute cardiac disease, especially if the dilemma of care futility arises, though these discussions did take place in some of the patients in our study. Unfortunately, we lack information regarding functional status: frailty (e.g., Clinical Frailty Status) or disability (ADL). We have reported the prevalence of “debilitated” patients, defined as at least one impairment in physical activities of daily living, instead. Finally, we acknowledge that the analytical exploration of outcome prediction is limited due the fact that the sample of nonagenarians is relatively small to perform a multivariate analysis.

## Conclusion

With increasing life expectancy, the prevalence of nonagenarians with acute cardiovascular disease will continue to increase. Thus, studies investigating this highly vulnerable population to generate evidence-based care are of paramount importance. In the current study, nonagenarian patients had more comorbidities and higher in-hospital mortality when compared with younger patients. Nevertheless, when treated with current standard of care including timely reperfusion when indicated, our nonagenarian population had better than expected outcomes after admission to the ICCU in comparison with previously published data. Further studies to create evidence-based knowledge to support and guide optimal management in these patients are warranted.

## Data Availability

The datasets used and/or analysed during the current study are available from the corresponding author on reasonable request.

## List of Abbreviations

CDV: Cardiovascular disease
ACS: Acute coronary syndrome
HF: Heart failure
VTE: Venous thromboembolism
ICCU: Intensive coronary care unit
ESC: European society of cardiology
STEMI: ST-segment elevation myocardial infarction
NSTEMI: Non-ST-segment elevation myocardial infarction
TTE: Transthoracic echocardiography
TEE: Transesophageal echocardiography
LVEF: Left ventricular ejection fraction
ARF: Acute renal failure
TAVI: Transcatheter aortic valve implantation
TEER: Transcatheter edge-to-edge repair
TEER: Transcatheter edge-to-edge repair
